# Species- and context-dependent responses of green lacewings suggest a complex ecological role for methyl salicylate (Neuroptera: Chrysopidae)

**DOI:** 10.1038/s41598-025-96730-z

**Published:** 2025-04-14

**Authors:** Sándor Koczor, Ferenc Szentkirályi, József Vuts, John C. Caulfield, David M. Withall, John A. Pickett, Michael A. Birkett, Miklós Tóth

**Affiliations:** 1 Plant Protection Institute, HUN-REN Centre for Agricultural Research, Budapest, Hungary; 2 Protecting Crops and the Environment, Rothamsted Research, Harpenden, Hertfordshire UK; 3 School of Chemistry, Cardiff University, Cardiff, UK

**Keywords:** *Chrysopa*, *Chrysoperla*, Methyl salicylate, Attraction, Synergism, Entomology, Chemical ecology

## Abstract

Herbivore-induced plant volatiles (HIPVs) are important semiochemicals in multitrophic plant-insect interactions, attracting natural enemies of phytophagous insects. Numerous studies have reported attraction of natural enemies to the HIPV methyl salicylate, including green lacewings. However, previous reports provide conflicting data, as some studies report significant attraction, whereas others found moderate or no attraction, even for the same taxa. In the current study, we conducted field experiments in Hungary with methyl salicylate and known attractants for *Chrysoperla* spp. and *Chrysopa* spp. lacewings. Both males and females of *Chrysoperla carnea* species complex were attracted to methyl salicylate, but to a much lesser extent compared to phenylacetaldehyde, a previously known floral attractant. When presented in combination, methyl salicylate showed a synergistic effect, confirming results of previous research performed in Hungary. On the other hand, methyl salicylate did not attract *Chrysopa formosa*. When tested in combination with known sex attractants for *C. formosa* males, it synergized male attraction to the aphid sex pheromone component (1*R*,4a*S*,7*S*,7a*R*)-nepetalactol; however, in combination with squalene, no synergistic effect was found. The results suggest that the responses of green lacewings to methyl salicylate are context-dependent, and the ecological role of the compound may be more complex than previously expected.

**Supplementary Information:**

The online version contains supplementary material available at 10.1038/s41598-025-96730-z.

## Introduction

Plant volatile organic compounds (VOCs) can act as semiochemicals of crucial importance in plant-insect relationships, for instance determining attraction or repellence^1^. They also play a key role in multitrophic interactions when released as herbivore-induced plant volatiles (HIPVs), by attracting insect natural enemies^2^. The HIPV methyl salicylate has been extensively studied as a kairomone for natural enemies of pest arthropods, with potential application in environmentally friendly agricultural practices (e.g^3^). Nevertheless, results to date are ambiguous, with some studies reporting successful application in pest management^4^, whilst others report lack of effect^5^.

Green lacewings (Neuroptera: Chrysopidae) comprise a species-rich family, with more than 1400 taxa described worldwide^6^. Their larvae hunt for soft-bodied pests, primarily Sternorrhyncha, including aphids^7^ and scale insects^8^. On the other hand, adults are generally not predatory, except for a few taxa (e.g. *Chrysopa* spp.)^7^.

Green lacewings are often inhabitants of agroecosystems such as arable field crops^9^, fruit orchards^10^ and forest ecosystems^11^. Some lacewing species are of special significance in biological control^12,13^, numerous studies have thus dealt with their attraction to semiochemicals^14^, including the HIPV methyl salicylate. Nevertheless, the results are controversial, as some studies report significant attraction^15^, whereas others moderate or no attraction^16^ even for the same chrysopid taxa. Methyl salicylate has also been found to have a synergistic effect in combination with attractants, such as iridodial^16,17^, 2-phenylethanol^18^ and phenylacetaldehyde^19^.

The aim of the current study was to clarify the activity of methyl salicylate in field conditions in comparison to, as well as in combination with, other, previously published attractants for *Chrysopa* species and common green lacewings (*Chryoperla carnea* complex). Tested compounds included the aphid sex pheromone component (1*R*,4a*S*,7*S*,7a*R*)-nepetalactol and squalene, both known to attract *Chrysopa* spp^16,20–22^, as well as phenylacetaldehyde, a known attractant for the *C. carnea* complex^23^.

## Materials and methods

### Preparation of baits

Methyl salicylate, phenylacetaldehyde and squalene (≥ 95% purity as per the manufacturer) were obtained from Sigma-Aldrich Kft. (Budapest, Hungary). (1*R*,4a*S*,7*S*,7a*R*)-Nepetalactol was synthesized as follows:

To a solution of *Nepeta cataria* essential oil (1.00 g) in methanol (20 ml), cooled to 0 ^o^C, was added sodium borohydride (498 mg, 13.2 mmol) and the mixture stirred for 60 min. The reaction was quenched with the addition of deionized water (10 ml) before being extracted with diethyl ether (3 × 20 ml). The combined organic layers were washed with water (3 × 20 ml), dried using anhydrous magnesium sulphate (MgSO_4_) and concentrated under vacuum. The crude product was purified on silica gel (20% diethyl ether in petroleum ether bp 40–60 ^o^C) to give (1*R*,4a*S*,7*S*,7a*R*)-nepetalactol as a colourless oil (632 mg). Spectroscopic analysis was consistent with previously reported data^24^.

For experiments 1, 2 and 3, compounds and combinations of compounds were formulated into 0.7 mL polyethylene vials (PE vials) with lid (No. 730, Kartell Co., Italy). The PE vial dispenser was chosen as it performed well in our previous experiments on *Chryopa* spp^25^.

For experiments 1 and 2, (1*R*,4a*S*,7*S*,7a*R*)-nepetalactol, methyl salicylate and their combinations and for experiment 3, (1*R*,4a*S*,7*S*,7a*R*)-nepetalactol, squalene and their combination with methyl salicylate were formulated into PE vials (Table 1). In order to provide a more comprehensive picture on responses of green lacewings, methyl salicylate, nepetalactol and their combinations were also tested in different doses in PE vial dispensers (Table 1).

**Table 1:** Treatments of field experiments. In experiments 1,2,3 polyethylene vial, whereas in experiment 4 polyethylene bag dispensers were used.

Treatments	Exp. 1	Exp. 2	Exp. 3	Exp. 4
Methyl salicylate	25 mg	50 mg	–	100 mg
Nepetalactol	25 mg	50 mg	100 mg	–
Nepetalactol + methyl salicylate (1:1)	25 + 25 mg	50 + 50 mg	100 + 100 mg	–
Nepetalactol + methyl salicylate (5:1)	–	50 + 10 mg	–	–
Nepetalactol + methyl salicylate (1:5)	–	10 + 50 mg	–	–
Phenylacetaldehyde	–	–	–	100 mg
Phenylacetaldehyde + methyl salicylate	–	–	–	100 + 100 mg
Squalene	–	–	100 mg	–
Squalene + methyl salicylate	–	–	100 + 100 mg	–
No bait	No bait	No bait	No bait	No bait

For experiment 4, methyl salicylate, phenylacetaldehyde and their combination were formulated into polyethylene bag (PE bag) dispensers (Table 1). These consisted of a 1 cm piece of dental roll (Celluron, Paul Hartmann AG, Heidenheim, Germany) put into a polyethylene bag (ca. 1.0 × 1.5 cm) made of 0.02 mm linear polyethylene foil (FS471-072, Phoenixplast BT, Pécs, Hungary). The PE bag dispenser was chosen as it performed well in our previous research on *Chryoperla* spp^22,25^. Based on our previous experiences with the PE bag dispenser, loads of individual compounds were kept at 100 mg (Table 1).

The lids of the PE vial dispensers were closed, PE bag dispensers were heat-sealed and both dispenser types were attached to 8 × 1 cm plastic handles for easy handling when assembling the traps. In the field experiments, PE bag dispensers were replaced at 3-4-week intervals, and PE vial dispensers were replaced at 4-5-week intervals, as previous experience showed that they did not lose their attractiveness during this period^22,25^.

For storage, all baits used in the experiments were wrapped singly in pieces of aluminium foil and stored at − 18 °C until used.

### Field experiments

Field experiments were performed at Halásztelek (Pest county, Central Hungary) in a mixed orchard (coordinates 47°21’9"N, 19° 0’20"E), using CSALOMON VARL + funnel traps (Supplementary Fig. 1, produced by Plant Protection Institute, HUN-REN Centre for Agricultural Research, Budapest, Hungary), which proved to be suitable for catching green lacewings in previous studies^21,22,25^. A small piece (1 × 1 cm) of household anti-moth strip (Chemotox, Sara Lee; Temana Intl. Ltd, Slough, UK; active ingredient 15% dichlorvos) was placed in the containers to kill captured insects.

Experiments were run in a randomized complete block design; one replicate of each treatment was incorporated into a block, so that individual treatments were 5–8 m apart in a randomized arrangement. To avoid positional effects, trap positions were changed on a fortnightly basis. Details of field experiments:

Experiment 1: The aim of this experiment was to test attraction of green lacewings to methyl salicylate, (1*R*,4a*S*,7*S*,7a*R*)-nepetalactol and their combination. Treatments included methyl salicylate only, (1*R*,4a*S*,7*S*,7a*R*)-nepetalactol only, methyl salicylate + (1*R*,4a*S*,7*S*,7a*R*)-nepetalactol and unbaited traps (Table 1). The experiment was run from 15th June to 10th September 2018, with 5 replicates.

Experiment 2: The aim of this experiment was to test attraction of green lacewings to methyl salicylate, (1*R*,4a*S*,7*S*,7a*R*)-nepetalactol and their combinations in different ratios. Treatments included methyl salicylate only, (1*R*,4a*S*,7*S*,7a*R*)-nepetalactol only, methyl salicylate + (1*R*,4a*S*,7*S*,7a*R*)-nepetalactol (in 1:1, 5:1 and 1:5 ratios) and unbaited traps (Table 1). The experiment was run from 18th July to 19th September 2019, with 5 replicates.

Experiment 3: The aim of this experiment was to test attraction of green lacewings to (1*R*,4a*S*,7*S*,7a*R*)-nepetalactol, squalene and their combination with methyl salicylate. Treatments included (1*R*,4a*S*,7*S*,7a*R*)-nepetalactol only, squalene only, (1*R*,4a*S*,7*S*,7a*R*)-nepetalactol + methyl salicylate, squalene + methyl salicylate and unbaited traps (Table 1). The experiment was run from 30th May to 11th September 2017, with 5 replicates.

Experiment 4: The aim of this experiment was to test attraction of green lacewings to methyl salicylate, phenylacetaldehyde and their combination. Treatments included methyl salicylate only, phenylacetaldehyde only, methyl salicylate + phenylacetaldehyde and unbaited traps (Table 1). The experiment was run from 5th July to 13th September 2022, with 5 replicates.

Traps were inspected on a weekly basis, catches were brought to the laboratory, where collected green lacewings were sexed and determined to species. The determination of lacewing species was based on the following taxonomic works: *Chrysoperla* spp: Henry et al.^26–28^; *Chrysopa gibeauxi*: Tillier et al.^29^; *Apertochrysa prasina* group:^30^. All other chrysopid species were determined according to Aspöck et al.^31^.

### Statistics

As *Chrysoperla lucasina* and *Chrysoperla pallida* were found in relatively low numbers in the experiments and previous studies did not report remarkable differences in the chemical ecology of these species^32^, *C. carnea* complex was treated as a unit in analysis of the results. Experimental data were calculated of weekly catches of individual traps. Weeks with no or very low catches, accounting for less than 5% of total catches of the respective experiment, were excluded from the statistical analysis. Catch data were tested for normality by Shapiro-Wilk test and since data were not normally distributed, nonparametric tests were used. Catch data were analyzed by Kruskal-Wallis test, and differences between treatments were evaluated by pairwise Wilcoxon test with Benjamini-Hochberg correction^33^. Statistical procedures were conducted using the software R^34^.

## Results

A total of 12 chrysopid species were recorded during the field experiments (Table 2); however, only catches of *C. formosa* and *C. carnea* complex were sufficient for statistical analysis.

**Table 2:** The number of green lacewing (Chrysopidae) species caught in the field experiments.

Species	Exp. 1	Exp. 2	Exp. 3	Exp. 4
*Chrysopa dorsalis* Burmeister, 1839	0	1	0	0
*Chrysopa formosa Brauer*,* 1851*	106	332	139	0
*Chrysopa gibeauxi* Leraut, 1989	0	2	0	0
*Chrysopa pallens* (Rambur, 1838)	9	6	1	0
*Chrysopa perla* (Linnaeus, 1758)	2	3	1	0
*Chrysoperla carnea* species complex:	25	43	43	250
*Chrysoperla carnea* (Stephens 1836)	23	38	31	197
*Chrysoperla lucasina* (Lacroix 1912)	1	4	5	42
*Chrysoperla pallida* Henry et al. 2002	1	1	7	11
*Nineta flava* (Scopoli, 1763)	0	0	1	0
*Peyerimhoffina gracilis* (Schneider, 1851)	0	0	1	0
*Apertochrysa prasina* group	0	2	1	5

**Fig. 1:**
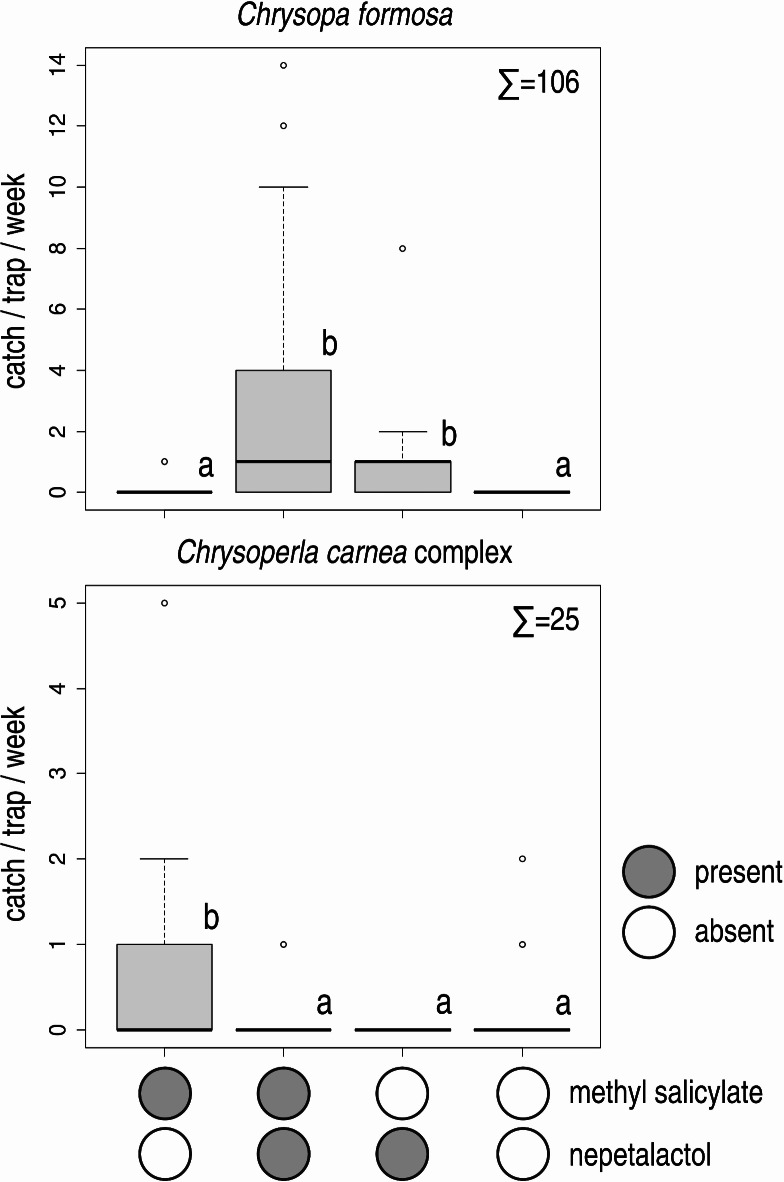
Catches of *Chrysopa formosa* and *Chrysoperla carnea* species complex in funnel traps baited with methyl salicylate, (1*R*,4a*S*,7*S*,7a*R*)-nepetalactol, their combination and in unbaited traps (Experiment 1). Catches marked with the same letter are not significantly different within one diagram (Kruskal–Wallis test, followed by pairwise comparisons by Wilcoxon rank sum test with Benjamini-Hochberg correction at *p* = 0.05) Σ = total catch of the respective species in the experiment.

In Experiment 1, treatments containing (1*R*,4a*S*,7*S*,7a*R*)-nepetalactol caught more *C. formosa* than those baited with methyl salicylate only and unbaited traps, the latter two not differing significantly (Fig. 1). Traps baited with (1*R*,4a*S*,7*S*,7a*R*)-nepetalactol + methyl salicylate caught higher numbers of *C. formosa* than those baited with (1*R*,4a*S*,7*S*,7a*R*)-nepetalactol only, this difference being marginally significant (*p* = 0.086) (Fig. 1). The vast majority (97.17%) of *C. formosa* caught were males.

Traps baited with methyl salicylate caught more *C. carnea* complex than all other treatments and unbaited traps, which did not differ from each other (Fig. 1). 60% of individuals caught were females.

**Fig. 2:**
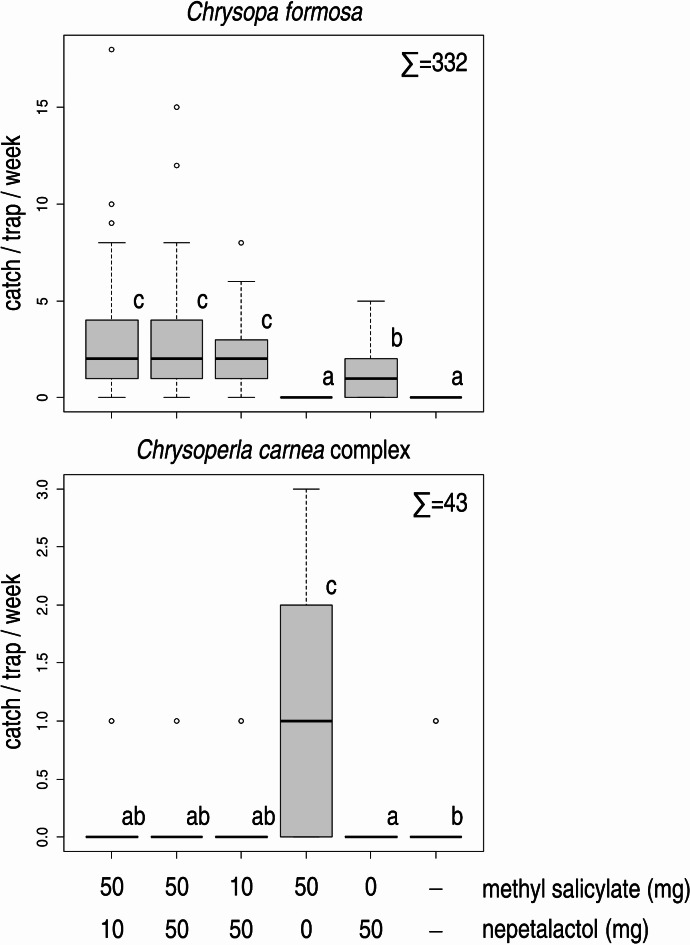
Catches of *Chrysopa formosa* and *Chrysoperla carnea* species complex in funnel traps baited with methyl salicylate, (1*R*,4a*S*,7*S*,7a*R*)-nepetalactol, their combination (in 1:1, 5:1 and 1:5 ratios) and unbaited traps (Experiment 2). Catches marked with the same letter are not significantly different within one diagram (Kruskal-Wallis test, followed by pairwise comparisons by Wilcoxon rank sum test with Benjamini-Hochberg correction at *p* = 0.05) Σ = total catch of the respective species in the experiment.

In Experiment 2, only treatments containing (1*R*,4a*S*,7*S*,7a*R*)-nepetalactol caught more *C. formosa* than unbaited traps. Adding methyl salicylate in different ratios to (1*R*,4a*S*,7*S*,7a*R*)-nepetalactol resulted in more individuals caught than by (1*R*,4a*S*,7*S*,7a*R*)-nepetalactol only (Fig. 2), the combinations not differing significantly from each other. Almost exclusively male *C. formosa* were caught (female ratio: 0.3%).

On the other hand, traps baited with methyl salicylate on its own caught more *C. carnea* complex lacewings than all other treatments and unbaited traps (Fig. 2). The majority (86.05%) of individuals caught were females.

In Experiment 3, for single compounds, traps baited with (1*R*,4a*S*,7*S*,7a*R*)-nepetalactol or squalene attracted more *C. formosa* than unbaited traps, their catches not differing significantly (Table 3). On the other hand, for combinations, (1*R*,4a*S*,7*S*,7a*R*)-nepetalactol + methyl salicylate caught significantly more individuals than all other treatments, whereas catches of squalene + methyl salicylate did not differ from those of unbaited traps. Almost exclusively males were caught (97.12%).

**Table 3:** Mean catches of *Chrysopa formosa* and *Chrysoperla carnea* species complex in traps baited with nepetalactol, squalene, their combination with methyl salicylate and in unbaited traps. Treatments marked with the same letter in a column are not significantly different at *p* = 5% by Kruskal–Wallis test, pairwise comparison by Wilcoxon test with Benjamini-Hochberg correction.

	*Chrysopa formosa*	*Chrysoperla carnea* complex
Treatment	Mean ± SE	Mean ± SE
Nepetalactol	0.34 ± 0.13 c	0.05 ± 0.05 a
Nepetalactol + methyl salicylate	2.06 ± 0.28 d	0.1 ± 0.07 a
Squalene	0.14 ± 0.05 bc	0.15 ± 0.08 a
Squalene + methyl salicylate	0.06 ± 0.03 ab	1.45 ± 0.45 b
No bait	0 ± 0 a	0.05 ± 0.05 a

For the *C. carnea* complex, squalene + methyl salicylate caught more individuals than all other treatments, catches of which not differing from those of unbaited traps (Table 3). Both males and females were caught (female ratio: 32.56%).

**Fig. 3:**
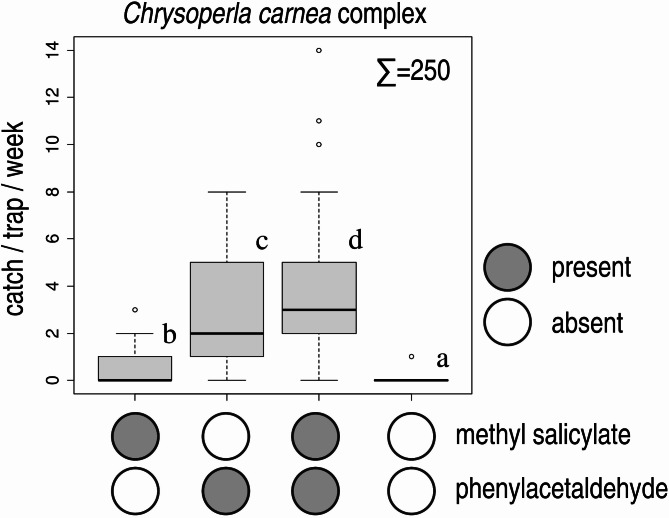
Catches of *Chrysoperla carnea* species complex in funnel traps baited with methyl salicylate, phenylacetaldehyde, their combination and in unbaited traps (Experiment 4). Catches marked with the same letter are not significantly different within one diagram (Kruskal-Wallis test, followed by pairwise comparisons by Wilcoxon rank sum test with Benjamini-Hochberg correction at *p* = 0.05) Σ = total catch in the experiment.

In Experiment 4, all baited treatments caught more *C. carnea* complex than unbaited traps. Traps baited with phenylacetaldehyde caught more *C. carnea* complex than those baited with methyl salicylate. The combination of phenylacetaldehyde + methyl salicylate caught more individuals than all other treatments (Fig. 3). A large percentage (62.40%) of individuals caught were females. No *C. formosa* were caught in the experiment (Table 2).

## Discussion

Our results show considerable differences in the responses of green lacewings to the HIPV methyl salicylate. Whereas both sexes of *C. carnea* complex were weakly attracted to this compound, predatory adults of *C. formosa* were not attracted at all, despite its potential ecological relevance as an HIPV indicating the presence of prey for the larvae and for the adults themselves. Methyl salicylate showed a synergistic effect in combination with phenylacetaldehyde, a known floral attractant for the *C. carnea* complex, and it synergized attraction of *C. formosa* males to the sex attractant (1*R*,4a*S*,7*S*,7a*R*)-nepetalactol, but not to squalene, another sex attractant.

Our experiments show that methyl salicylate attracts both male and female adults of the *C. carnea* complex, which effect was significant in both dispenser types and in all tested doses. However, the known floral attractant phenylacetaldehyde^23^ clearly outperformed methyl salicylate, attracting significantly more *C. carnea* complex adults, which suggests that methyl salicylate has only a weak attractive effect as a single stimulus. Phenylacetaldehyde is possibly attractive to adult *C. carnea* complex as a floral cue from potential food sources, because adults are flower visitors, feeding on pollen and nectar^7^. On the other hand, methyl salicylate as a HIPV may also be a valuable cue to ovipositing females, because the larvae are predatory^7^. Nevertheless, it is important to note that methyl salicylate is a floral volatile in several plant families^35^, it thus may also be an important cue for flower visiting adults. In the experiments, a large percentage of attracted *C. carnea* complex were females.

Addition of methyl salicylate to phenylacetaldehyde significantly increased the number of attracted *C. carnea* complex adults. The results are in line with previous studies on *Chrysoperla* spp., where methyl salicylate synergized attraction to phenylacetaldehyde, whereas it was clearly outperformed by phenylacetaldehyde-based combinations and showed negligible effect when presented on its own^19^. These results underline that combined stimuli may elicit stronger behavioural responses than single ones^36^.

On the other hand, the addition of (1*R*,4a*S*,7*S*,7a*R*)-nepetalactol to methyl salicylate significantly decreased attraction of *C. carnea* complex adults, but no such effect was observed for the combination of methyl salicylate + squalene in the present study. Similar observations were made on combinations of (1*R*,4a*S*,7*S*,7a*R*)-nepetalactol and squalene with a ternary floral bait attractive to *C. carnea* complex^21,22,25^.

Predatory adults of *C. formosa* were not attracted to methyl salicylate in our experiments, irrespective of dispenser type or dose, although the species was present in the orchard as confirmed by catches of known attractants. This was unexpected, because attraction of both sexes of *Chrysopa nigricornis* to methyl salicylate in a different geographic region (North America) has been reported^15^.

Addition of methyl salicylate to (1*R*,4a*S*,7*S*,7a*R*)-nepetalactol, a common aphid sex pheromone component and a known attractant for *C. formosa* males^20,21^, resulted in increased catches, synergizing the activity of the sex attractant. The different ratios of (1*R*,4a*S*,7*S*,7a*R*)-nepetalactol and methyl salicylate did not differ in their activity in the tested dose range (5-fold differences); however, all combinations resulted in increased catches as compared to (1*R*,4a*S*,7*S*,7a*R*)-nepetalactol alone. This indicates that the presence of both stimuli is important, but they may elicit attraction of males in a wider range of ratios. It is unclear why no female *C. formosa* were attracted to any combinations in our experiments, i.e. why almost exclusively males were caught. To date, no attractants for female *C. formosa* are known.

Interestingly, when methyl salicylate was added to squalene, another attractant for *C. formosa* males^22^, no synergistic effect was observed, suggesting a different ecological background for (1*R*,4a*S*,7*S*,7a*R*)-nepetalactol and squalene. To date, the role of these compounds in the chemical ecology of *Chrysopa* spp. is not clear. It was suggested that (1*R*,4a*S*,7*S*,7a*R*)-nepetalactol is a potential precursor for the production of the male-produced, male-attracting pheromone (1*R*,2*S*,5*R*,8*R*)-iridodial in the nearctic *Chrysopa oculata*^37^. The authors proposed that males consume oviparous aphids to sequester the precursor; however, adult *C. formosa* are not found in late season when oviparous aphids are present^38^. Furthermore, no such male-produced pheromone is known for *C. formosa* to date, studies so far only confirming the production of defensive chemicals^39^.

The current results underline the species- and context-dependent responses of green lacewings to methyl salicylate. Context-dependence in response to methyl salicylate was also observed in aphids, where summer migrants of the bird-cherry oat aphid, *Rhopalosiphum padi,* were repelled by methyl salicylate as a semiochemical released by the winter host^40^.

Despite their importance as biological control agents, knowledge on the chemical ecology of only a few percent of green lacewing species exists^14^. Studies on other species may shed light on the ecological and evolutionary complexity of Chrysopidae chemical ecology, with possibly highly interesting contributions to the chemical ecology of insects in general.

## Electronic supplementary material

Below is the link to the electronic supplementary material.

Supplementary Material 1

## Data Availability

The datasets used and/or analysed during the current study available from the corresponding author on reasonable request.
