# Supplementary material for: Species- and context-dependent responses of green lacewings suggest a complex ecological role for methyl salicylate (Neuroptera: Chrysopidae)
Source: Sci Rep. 2025 Apr 14;15:12777. doi: 10.1038/s41598-025-96730-z (PMC11997097; doi:10.1038/s41598-025-96730-z)
Supplement: Supplementary file 1 — Supplementary Material 1 [file 41598_2025_96730_MOESM1_ESM.pdf]

Electronic Supplementary Material

Title: Species- and context-dependent responses of green lacewings suggest a complex ecological role for methyl salicylate (Neuroptera: Chrysopidae)
Journal: Scientific Reports

Authors:
Sándor Koczor*[1], Ferenc Szentkirályi[1], József Vuts[2], John C. Caulfield[2], David M. Withall[2], John A. Pickett[3], Michael A. Birkett[2], Miklós Tóth[1]
[1] Plant Protection Institute, HUN-REN Centre for Agricultural Research, Budapest, Hungary
[2] Protecting Crops and the Environment, Rothamsted Research, Harpenden, Hertfordshire, United Kingdom
[3] Cardiff University, School of Chemistry, Cardiff, United Kingdom
*corresponding author: koczor.sandor@atk.hun-ren.hu

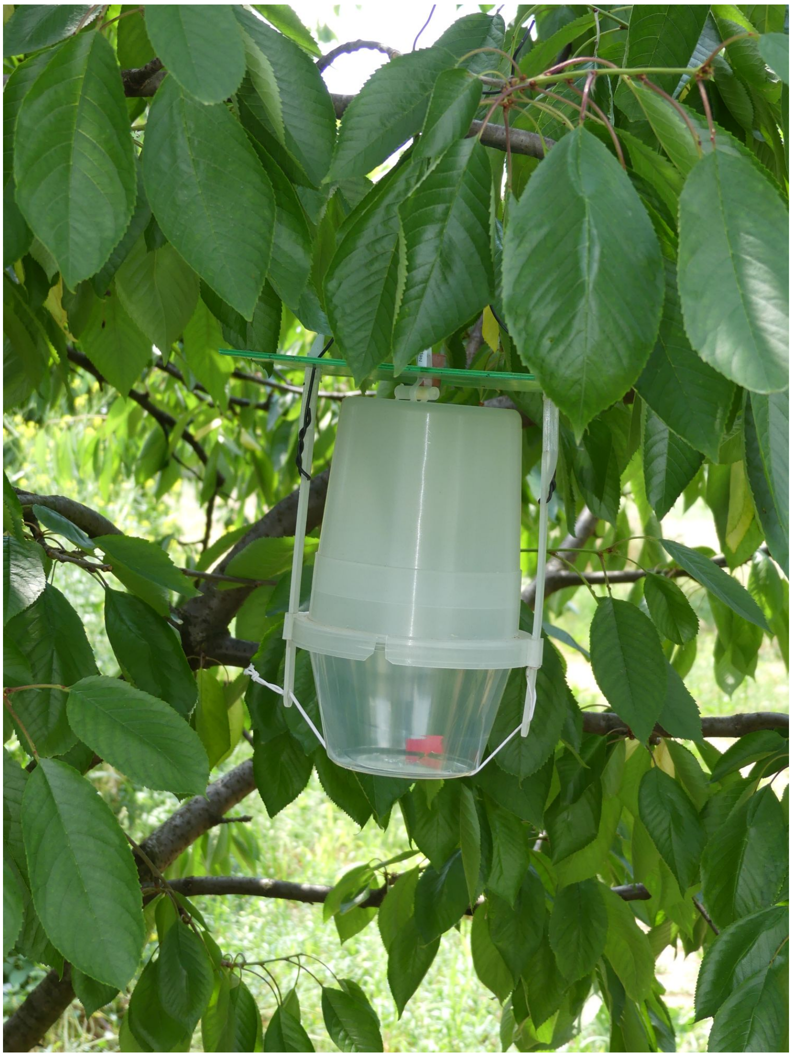

Supplementary Fig. 1: The CSALOMON VARL trap used in the experiments
